# Health-related quality of life before and during chemotherapy in patients with early-stage breast cancer

**DOI:** 10.3332/ecancer.2020.1007

**Published:** 2020-01-27

**Authors:** Monique Binotto, Tomás Reinert, Gustavo Werutsky, Facundo Zaffaroni, Gilberto Schwartsmann

**Affiliations:** 1 Postgraduate Program in Clinical Research, Hospital de Clínicas de Porto Alegre, HCPA, Porto Alegre, RS 90035-007, Brazil; 2 Centro de Pesquisa da Serra Gaúcha, CEPESG, Caxias do Sul, RS 95020-450, Brazil; 3 Latin American Cooperative Oncology Group, LACOG, Porto Alegre, RS 90619-900, Brazil; a https://orcid.org/0000-0002-5799-5390; b https://orcid.org/0000-0003-4715-1415; c https://orcid.org/0000-0001-6271-105X; d https://orcid.org/0000-0002-7850-1644

**Keywords:** health-related quality of life, breast cancer, patient-reported outcomes, cancer

## Abstract

**Objectives:**

Identify the main changes in the health-related quality of life (HRQoL) of women diagnosed with breast cancer (BC) undergoing chemotherapy.

**Methods:**

Prospective cohort study that included 33 women diagnosed with clinical stages I–III BC and who underwent adjuvant chemotherapy. HRQoL was assessed using the EORTC QLQ-C30 and EORTC QLQ-BR23 instruments 1 week before the start of chemotherapy and during the third month of chemotherapy.

**Results:**

There was a decline in the HRQoL scores of patients during treatment. Therefore, chemotherapy alters the patient’s perceptions of their HRQoL since there is a decrease in global health status/quality of life (QoL) and functional scales such as physical functioning, role functioning, emotional functioning, social functioning, body image, sexual function and sexual enjoyment. We also observed an increase in side effects related to the systemic therapy, fatigue, nausea and vomiting, insomnia, appetite loss and diarrhoea, despite a decrease in breast symptoms and arm symptoms.

**Conclusions:**

HRQoL was negatively affected during chemotherapy. Even though HRQoL assessment is a useful method for optimising patients’ care, its implementation into clinical practice remains a challenge. Since side effects are very often underestimated, we consider that the evaluation of HRQoL parameters should be done for BC patients treated with chemotherapy.

## Background

According to current projections, the number of new cases of cancer is increasing and will grow from 14 million in 2012 to an annual global number of 22 million by 2030 [1]. Breast cancer (BC) is the second most common cancer in the world in terms of new cases (1.7 million cases) and ranks fifth place as the cause of death [2]. Given the increasing survival rates after BC treatment, there is a greater emphasis on enhancing health-related quality of life (HRQoL) during and after oncologic therapies. Systemic therapies are associated with significant benefits in terms of reducing the risk of BC recurrence [3] and are also associated with negative effects on HRQoL of survivors [4]. Additionally, given the fact that BC is being increasingly diagnosed in earlier stages as a consequence of screening programmes, the number of women receiving curative-intent adjuvant chemotherapy is also increasing.

It is necessary to consider the symptomatology and the adverse effects profile of different types of treatments that impact the patient’s HRQoL. Chemotherapy, for example, causes significant side effects in women with BC such as fatigue, febrile neutropenia, depression, dyspnoea, pain, nausea and vomiting [5]. Besides, cancer-related sequelae can include emotional distress like uncertainty or fear of recurrence and posttraumatic stress symptoms, pain and limitations in the ability to work [6]. Although there is a consensus in the literature that chemotherapy is the treatment that most impacts the HRQoL, specific real-world data (RWD) about the impact of adjuvant chemotherapy in HRQoL of BC patients are not clear.

The concept of quality of life (QoL) can be defined as the individual’s perception of his position in life in the context of culture and value systems in which they live and in relation to their goals, expectations, standards and concerns [7]. When this concept is restricted to health perceptions, the term is called HRQoL. This term is a multi-domain concept, which represents the general perception of the patient focusing on the effect of disease and treatment on other aspects of life [8]. Therefore, the term HRQoL is used to include in this assessment those aspects that are generally not included in the health context (such as income, freedom and quality of the environment). Therefore, focusing on the assessment of HRQoL means to evaluate almost all aspects of life that are health-related [9].

Therefore, it is essential to understand the patients’ needs in order to improve HRQoL and to stabilise mental, social and physical health, in addition to the management of specific signs and symptoms throughout the treatment. In this context, the purpose of this study was to evaluate the HRQoL in BC patients and to compare the patterns before and during chemotherapy.

## Methods

We conducted a prospective cohort study to evaluate HRQoL in 33 Brazilian BC patients who performed the first oncological consultation at a private oncology clinic in the city of Caxias do Sul, RS, Brazil. The inclusion criteria were women ≥ 18 years of age, histologically confirmed invasive stages I–III BC who were treated with breast surgery and received adjuvant or neoadjuvant treatment with an anthracycline and/or taxane-based chemotherapy.

The evaluation of patients HRQoL used the EORTC QLQ-C30 (European Organisation for Research and Treatment of Cancer Quality of Life Core Questionnaire) and EORTC QLQ-BR23 (EORTC BC-specific Quality of Life Questionnaire) [10]. All of these instruments are validated, translated to Portuguese, standardised and self-administrative [11]. Patients completed the questionnaires in two periods, i.e. 1 week before the beginning of the chemotherapy treatment (baseline) and in the third month of treatment, approximately in the fourth chemotherapeutic cycle (3-month follow-up). The following information were collected from medical records such as sociodemographic data (educational level and marital status), habits (smoking and alcoholism), menopausal status, family history of cancers and performance status – evaluated no more than 2 weeks before enrolment into the study using the Eastern Cooperative Oncology Group Scale [12].

We evaluated the questionnaire according to standardised methodology recommended by the EORTC Group. Data analysis was performed using SAS version 9.4. The sociodemographic and clinical-epidemiological results were described by means, standard deviation, median, minimum, maximum, first quartile (1° Q) and third quartile (3° Q) or using of absolute and relative frequencies. To analyse the paired data in the two follow-up times, the Wilcoxon test was used, considering statistically significant those results whose *p*-value (*p*) was ≤ 0.05. There were no adjustments for multiplicity.

This study was approved by the Research Ethics Committee (numbered 2.106.211, CAAE: 66288117.0.0000.5327). All the participants signed the Term of Free and Informed Consent, agreeing to participate in this research.

## Results

### Study population

Results concerning sociodemographic and clinical characteristics from the 33 patients included in the study are described in Tables 1 and 2. The patients had a median age of 51.4 years and were mostly married (75.8%), almost half of them studied until elementary or middle school (48.5%), the majority were non-smokers (72.7%) and non-alcoholic (81.8%). Regarding patients’ clinical characteristics, almost half of them were premenopausal in diagnosis (42.4%), and most of them had a family history of cancer (60.6%).

### HRQoL scores

**Figure 1.**
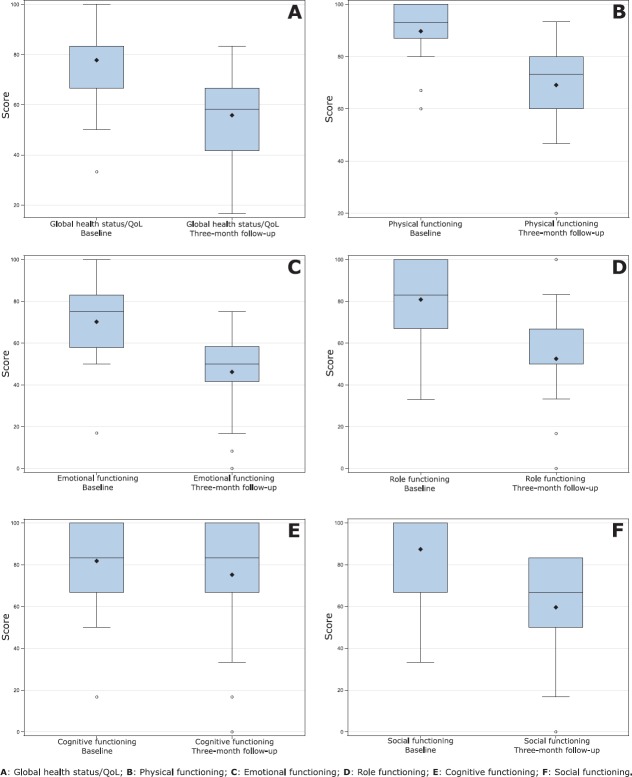
Comparison of HRQoL between study segments in global health status/QoL and functional scales (EORTC QLQ-C30).

With the use of EORTC QLQ-C30 questionnaire, a significant difference was observed between the evaluation performed before the start of the chemotherapy and the reassessment in the third month treatment (Table 3 and Figure 1). The scores decreased significantly in the global health status/QoL (*p* < 0.0001) and in the functional scales such as physical functioning (*p* < 0.0001), role functioning (*p* <0.0001), emotional functioning (*p* < 0.0001) and social functioning (*p* < 0.0001). In terms of symptoms, there was a significant increase in the scales of fatigue (*p* < 0.0001), nausea and vomiting (*p* = 0.0002), insomnia (*p* = 0.0017), appetite loss (*p* = 0.0098) and diarrhoea (*p* = 0.0241).

Significant alterations were also observed in the EORTC QLQ-BR23 questionnaire before and during chemotherapy (Table 4). Functional scales obtained lower scores in the second evaluation, performed during treatment, for the body image (*p* = 0.0005), sexual function (*p* < 0.0001) and sexual enjoyment (*p* = 0.0002). Symptom scales increased in systemic therapy side effects (*p* < 0.0001) and decreased of breast symptoms (*p* = 0.0040) and arm symptoms (*p* = 0.0253).

## Discussion

The evaluation of HRQoL can be very useful to measure the impact of treatment on health-disease perceptions, psychological issues, life satisfaction and patients’ well-being [13]. BC patients are at increased risk of treatment side effects on general HRQoL, e.g., physical conditions (fatigue, sleep disorders and pain) and psychological disorders (depression, anxiety, fear of recurrence, problems related to sexuality and body image) [6, 14]. Also, chemotherapy alters the patient’s perceptions of their HRQoL since there is an increase in symptoms and a decrease in functional scales [15–17].

This study is important because we performed an analysis with RWD from Brazilian BC patients. In addition, HRQoL analysed are rare outside of randomised clinical trials. Thus, patients analysed in this study may describe more realistically the changes in HRQoL triggered during chemotherapy. These data may help clinical oncologists to measure the magnitude of the effect that chemotherapy may have on potential biopsychosocial characteristics, in addition to the classic symptoms that chemotherapy triggers in patients.

In this study, the HRQoL of BC patients undergoing neoadjuvant and adjuvant chemotherapy is worse when comparing the period before the start of treatment with the one during chemotherapy. Global health status/QoL declined due to chemotherapy indicating that women with BC consider that their health status is deteriorated during treatment. Similarly, Leinert *et al* [15] showed that global health deteriorated during chemotherapy treatment also in the context of an increase in symptoms attributed to systemic treatment.

Additionally, BC patients have a high risk of developing alterations in their psychological functions, which also has a negative impact on HRQoL [18]. According to the literature [19, 20], there is a considerable decline in the scales of emotional functioning and body image during chemotherapy. It is believed that altered body image is a critical psychosocial issue for women with BC [21]. This can be explained because body image may be impacted by the patients’ perceptions about what others think, thereby affecting person’s sense of self-esteem. The global HRQoL, the low social and emotional functioning of BC patients were significantly associated with psychological variables [22]. Thus, it is understood that, in general, the severity of the symptoms is associated with adverse psycho-behavioural characteristics [23].

Among the women who received chemotherapy, a decrease was observed over time in breast and arm, which can be associated with the improvement and resolution of post-operative complications such as pain, function limitation of the upper limb and lymphedema. A similar result was found in the study by Winters *et al* [24], whose patients also reported improvements in locoregional symptoms over time. Moreover, there were no differences between the periods in cognitive functioning, pain, dyspnoea, constipation, financial difficulties and future perspective between the two evaluations.

These scales are about physical effort, sleep and help with basic needs and the ability to work or perform daily activities. This consequence is common in patients with BC due to disease- and treatment-related limitations in their functional status. Similar results are described in the literature [15, 16] since physical functioning is consistently better in the baseline compared to the end of treatment due to the development of fatigue [16]. Regarding the upset by hair loss scale, it is not possible to compare the segments. According to the EORTC Scoring Manual [25], the variation in the number of responses in EORTC QLQ-BR23 is predicted since scales upset by hair loss and sexual enjoyment are not applicable when the responses related to this scale are ‘no’. Even so, a high score on this scale represents that the symptom negatively impacted the patient’s HRQoL [25]. Therefore, alopecia is life altering and the patients considered these side effects distressing [21]. In this way, this alteration can develop a fear regarding the way it will be seen or judged by the others, causing the withdrawal from social life because it feels uncomfortable in public places [21, 26].

Several factors are associated with the social withdrawal of patients with BC. There is social stigmatisation about the disease, which can affect the relationship of the BC woman with other people. In our study, social functioning was impaired throughout chemotherapy, which means that physical condition and treatment interfered in the way a patient relates to his family and participates in social activity. Similar results were reported in other studies [16, 27], demonstrating the impact that chemotherapy treatment can have on social relations. On the other hand, larger social networks are related to higher HRQoL after a diagnosis of BC, when the patient has greater social support from family and friends [28]. It is understood that sexuality, after a cancer diagnosis, can be influenced by changes in hormone levels and changes in body image perception [29]. Our findings are in agreement with the study of Hall *et al* [30], which indicated that most of the systemic effects of chemotherapy tend to compromise women’s sexuality in the short- and long-term. The findings of increased systemic adverse effects (systemic therapy side effects, fatigue, nausea and vomiting, insomnia, appetite loss and diarrhoea) in patients treated with chemotherapy agreed with the broader toxicity results expected for the treatment.

Chemotherapy may also be responsible for exacerbating low-grade toxicities [31] such as diarrhoea, which may be sufficient to impair patients’ HRQoL. Besides, insomnia is also a common problem in cancer patients. The concomitant effect of chemotherapy on insomnia symptoms is mediated by a range of symptoms of oncologic therapy such as urinary symptoms, nausea, night sweats and digestive symptoms [32]. Changes in taste and smell often occur as a side effect of chemotherapy. These changes affect food behaviour, reducing overall food intake or restricting the intake of specific foods [33].

The current study has as a limitation due to the absence of more evaluations after the one realised at the 3 months of chemotherapy treatment. Thus, it was not possible to evaluate if the impact of chemotherapy on the HRQoL of these patients was reversible to the basal levels after a longer period. Despite this limitation, the objective of the study was reached the evaluation of the impact of chemotherapy on the patient’s HRQoL and the identification of possible factors that have an impact on the social and psychological parameters of the patient’s well-being. More studies should be done in a similar population in order to analyse the long-term HRQoL effects of adjuvant chemotherapy in BC patients. One additional limitation is that only patients treated in the private setting were included, and therefore, our study population may not represent adequately the patients treated in the public health system.

Additionally, it is important to emphasise that there is a variety of chemotherapeutic agents and combinations used for the treatment of BC and each regimen is associated with a specific adverse effect profile. All of our patients received standard anthracycline and taxane-based chemotherapy regimens. Therefore, our findings cannot be extrapolated to patients treated with different schemas such as Cyclophosphamide, Methotrexate, and 5-Fluorouracil (CMF) and capecitabine that usually have a milder side effects profile and probably impact HRQoL in a different way.

The findings of our study contribute to the knowledge about the needs of women with BC during chemotherapy treatment. The results show that the HRQoL of these patients is negatively altered during chemotherapy, and therefore, there is a need for interventions in oncology and research in this area. The implementation of HRQoL assessment in clinical practice has already been tested in a randomised controlled trial [34]. In this study, the routine assessment of cancer patients’ HRQoL indicated that is an effective approach for improves the quality of healthcare (with a positive impact on physician–patient communication). Despite the difficulties in implementing an HRQoL assessment programme in practice in Latin American countries, like Brazil, this parameter must be considered to provide chemotherapy every time for early BC given that short- and long-term impacts on QoL are often under estimated.

The period just after diagnosis and treatment is a critical point to the assessment of patient’s needs for coping and planning. Because of this, healthcare providers should give special attention to potential issues in the adjustment of the patient to the treatment. The quality of the information provided at this moment may improve patients’ sense of well-being [21]. Physicians and health’s professionals should screen patients often for systemic therapy side effects and use symptom scales. Screening can consider also the patient’s perceptions of global health status and QoL, physical functioning, role functioning, emotional functioning and social functioning. In this setting, basic communication skills and empathy are essential when conducting a psychosocial assessment.

It is important to understand patient and family goals in order to help the adjustment of the treatment around their priorities and ensure that we care for the whole person, considering perceptions about cancer care and well-being during treatment. Minimising treatment adverse effects and managing strategies to help the patient overcome this step are critical to the improvement of HRQoL. Using this information, we can tailor our treatment strategy to the needs of each patient.

## Conclusions

The HRQoL of BC patients is generally worse during the third month of chemotherapy when compared to the period before the start of treatment. Our findings are in agreement with what has been reported in the literature. Although the implementation of QoL assessment methodologies in the care routine is still a challenge, patients could have many benefits associated with the improvement of HRQoL and wellbeing during the treatment. Researchers and healthcare providers should continue to extend models for disseminating knowledge about HRQoL. Moreover, the analysis of HRQoL in the real-world setting is important and should be considered a priority in future research within this field.

## Conflicts of interest

The authors have no conflicts of interest to declare.

## Funding statement

The authors received no financial support for the research, authorship and/or publication of this article.

## Authors’ contributions

All the authors contributed to the elaboration of this paper and agreed with the content.

## Trial registration

Not applicable.

## Figures and Tables

**Table 1.** Patients’ characteristics.

Characteristics	*n* (%)
Age, mean (SD)	51.4 (11.8)
Marital status	
Married Single	25 (75.8) 8 (24.2)
Educational level	
Elementary or middle school High school College	16 (48.5) 12 (36.4) 5 (15.1)
Habits (smoke)	
Non-smoker Former smoker Current smoker	24 (72.7) 4 (12.1) 5 (15.2)
Habits (alcoholism)	
Non-alcoholic Former alcoholic	27 (81.8) 6 (18.2)

**Table 2.** Patients’ characteristics.

Characteristics	*n* (%)
Menopausal state	
Premenopausal Perimenopausal Postmenopausal	14 (42.4) 7 (21.2) 12 (36.4)
Family history of cancer	
No Yes (breast) Yes (breast and other) Yes (other)	13 (39.4) 4 (12.1) 7 (21.2) 9 (27.3)
ECOG performance status	
0 1	18 (54.5) 15 (45.5)
Mode of BC detection	
Screen detected Symptomatic Unknown	13 (39.4) 19 (57.6) 1 (3.0)

**Table 3.** Comparison of HRQoL between the study segments (EORTC QLQ-C30).

		N	Mean	Standard deviation	Median	Quartiles		*p*-value
						1°	3°	
*Global health status/QoL*								
Global health status/QoL	Baseline Three-month follow-up	33 33	77.77 55.82	16.23 16.067	83.30 58.33	66.70 41.70	83.30 66.70	<0.0001*
*Functional scales*								
Physical functioning	Baseline Three-month follow-up	33 33	89.73 69.09	11.58 15.44	93.00 73.33	87.00 60.00	100.00 80.00	<0.0001*
Role functioning	Baseline Three-month follow-up	33 33	80.82 52.53	19.10 23.24	83.00 50.00	67.00 50.00	100.00 66.67	<0.0001*
Emotional functioning	Baseline Three-month follow-up	33 33	70.21 46.21	19.42 20.95	75.00 50.00	58.00 41.67	83.00 58.33	<0.0001*
Cognitive functioning	Baseline Three-month follow-up	33 33	81.82 75.25	20.56 25.38	83.30 83.33	66.70 66.70	100.00 100.00	0.3050
Social functioning	Baseline Three-month follow-up	33 33	87.38 59.60	18.64 22.44	100.00 66.70	66.70 50.00	100.00 83.30	<0.0001*
*Symptom scales*								
Fatigue	Baseline Three-month follow-up	33 33	14.14 55.56	16.50 19.84	11.11 55.56	0.00 44.44	22.22 66.67	<0.0001*
Nausea and vomiting	Baseline Three-month follow-up	33 33	1.01 13.64	4.04 21.02	0.00 0.00	0.00 0.00	0.00 16.67	0.0002*
Pain	Baseline Three-month follow-up	33 33	27.78 32.32	27.85 26.98	16.67 33.33	0.00 16.67	50.00 50.00	0.3835
Dyspnoea	Baseline Three-month follow-up	33 33	7.07 14.14	18.18 26.39	0.00 0.00	0.00 0.00	0.00 33.33	0.2529
Insomnia	Baseline Three-month follow-up	33 33	28.28 52.53	32.41 31.21	33.33 33.33	0.00 33.33	33.33 66.67	0.0017*
Appetite loss	Baseline Three-month follow-up	33 33	7.07 22.22	18.18 29.66	0.00 0.00	0.00 0.00	0.00 33.33	0.0098*
Constipation	Baseline Three-month follow-up	33 33	18.18 31.31	28.98 35.30	0.00 33.33	0.00 0.00	33.33 66.67	0.0930
Diarrhoea	Baseline Three-month follow-up	33 33	1.01 9.09	5.80 17.23	0.00 0.00	0.00 0.00	0.00 16.67	0.0241*
Financial difficulties	Baseline Three-month follow-up	33 33	26.26 30.30	28.57 29.30	33.33 33.33	0.00 0.00	33.33 33.33	0.5582

* Statistically significant *p*-value from the Wilcoxon test

**Table 4.** Comparison of HRQoL between study segments. Specific questionnaire for BC (EORTC QLQ-BR23).

		N	Mean	Standard deviation	Median	Quartiles		*p*-value
						1°	3°	
*Functional scales*								
Body image	Baseline Three-month follow-up	33 33	90.66 66.41	14.99 31.49	100.00 75.00	91.67 50.00	100.00 100.00	0.0005*
Sexual functioning	Baseline Three-month follow-up	33 33	51.52 17.68	19.26 15.56	50.00 16.67	33.33 0.00	66.67 33.33	<0.0001*
Sexual enjoyment^†#^	Baseline Three-month follow-up	15 15	68.89 24.44	23.46 15.26	66.67 33.33	66.67 0.00	100.00 33.33	0.0002*
Future perspective	Baseline Three-month follow-up	33 33	32.32 39.39	33.83 35.80	33.33 33.33	0.00 0.00	66.67 66.67	0.4385
*Symptom scales*								
Systemic therapy side effects	Baseline Three-month follow-up	33 33	8.51 45.02	9.36 19.20	4.76 38.10	0.00 33.33	9.52 57.14	<0.0001*
Breast symptoms	Baseline Three-month follow-up	33 33	32.32 18.94	23.91 22.94	25.00 8.33	16.67 0.00	50.00 25.00	0.0040*
Arm symptoms	Baseline Three-month follow-up	33 33	34.34 20.20	30.22 25.83	22.22 11.11	11.11 0.00	55.56 22.22	0.0253*
Upset by hair loss^#^	Baseline Three-month follow-up	0 33	NA 61.62	NA 36.44	NA 66.67	NA 33.33	NA 100.00	-

* Statistically significant *p*-value from the Wilcoxon test

† Only 15 women with valid baseline and follow-up information (third month) were considered. NA, not applicable; there was no valid information available.

# According to the EORTC Scoring Manual [25], the variation in the number of responses in EORTC QLQ-BR23 is predicted since the fields ‘sexual enjoyment’ and ‘upset by hair loss’ do not apply when the responses related to these scales are ‘no’.
